# Fluoride: Is It Worth to be added in Pit and Fissure Sealants?

**DOI:** 10.5005/jp-journals-10005-1125

**Published:** 2012-02-24

**Authors:** AR Prabhakar, Prasanna T Dahake, OS Raju, N Basappa

**Affiliations:** Professor and Head, Department of Pedodontics and Preventive Dentistry, Bapuji Dental College and Hospital, Davangere-577004 Karnataka, India, e-mail: attiguppeprabhakar@gmail.com; Postgraduate Student, Department of Pedodontics and Preventive Dentistry, Bapuji Dental College and Hospital, Davangere, Karnataka India; Professor, Department of Pedodontics and Preventive Dentistry, Bapuji Dental College and Hospital, Davangere, Karnataka, India; Professor, Department of Pedodontics and Preventive Dentistry, Bapuji Dental College and Hospital, Davangere, Karnataka, India

**Keywords:** Dental caries, Pit and fissure sealants, Fluorides

## Abstract

**Background and objectives:** Fluoride is being used for the prevention of dental caries since a long time. Incorporation of fluoride in pit and fissure sealants has been found to reduce initiation and progression of pit and fissure caries. Authors conducted this study to evaluate and compare the effect of fluoride releasing pit and fissure sealants on the inhibition of demineralization of adjacent enamel and to reduce wall lesion frequency.

**Materials and methods:** A total of 60 caries-free human third molars were randomly assigned into three groups receiving conventional resin sealant without fluoride (Group A), fluoride releasing resin sealant (Group B), glass ionomer pit and fissure sealant (Group C). Fissure cavities of 5 × 2 × 1.5 mm were prepared on buccal surfaces of teeth using fissurotomy bur and sealants were applied onto the cavities.

The teeth were then thermocycled and exposed to acidified gelatin gel for 6 weeks to induce caries like lesions. A 150 μ m section was taken from each tooth and observed under polarized light microscope to measure the depth of advancing front of outer enamel lesion. The outer lesion depths of all three groups were compared.

**Results:** Enamel demineralization was least in glass ionomer pit and fissure sealant while the demineralization exhibited by nonfluoridated resin and fluoridated resin were comparable. Wall lesion frequency was found to be 0% in all groups.

**Conclusion and interpretation:** The glass ionomer pit and fissure sealant exhibited highest anticariogenic efficacy and hence can be advocated as a means of preventing dental caries.

**How to cite this article:** Prabhakar AR, Dahake PT, Raju OS, Basappa N. Fluoride: Is It Worth to be added in Pit and Fissure Sealants?. Int J Clin Pediatr Dent 2012;5(1):1-5.

## INTRODUCTION

Dental caries is the most prevalent chronic disease affecting the human race.^1^ Dental caries remains the singlemost common disease of childhood, occurring five to eight times more commonly than asthma, which is the second most common disease of childhood.^2^

Occlusal surface represents 12.5% of total surface of permanent dentition but accounts for more than 50% of caries in school children. Occlusal pits and fissures are the areas of caries initiation. Attempts are being made since a long time to prevent initiation and progression of pit and fissure caries by various means.^3^ Introduction of Bis-GMA has revolutionized the pit and fissure sealant treatment. Since then, many advancements have been made to improve their adhesive and mechanical properties. Pit and fissure sealants are an economical and adequate means for prevention of dental caries on occlusal surface of molars and premolars and to maintain dental health. Various other materials like flowable composites, glass ionomer cements, resin-modified glass ionomer cements, compomers and different types of bonding agents have also been used nowadays as pit and fissure sealants.

The topical and systemic fluorides are effective in reducing the smooth surface caries but are ineffective in preventing pits and fissures caries.^4^ Incorporation of fluorides in pit and fissure sealants has been found to play a promising role in the reduction of pit and fissure caries, thereby reducing overall caries incidence. Different types of fluoride releasing pit and fissure sealants can be used to protect smooth surfaces, hypoplastic enamel and areas around orthodontic brackets. Considering these advantages, use of sealants is advocated in various public health prevention measures and has to be proved successful.^5^

Hence, the present study was conducted to evaluate and compare the potential of fluoride releasing pit and fissure sealants on the inhibition of demineralization of adjacent enamel and to reduce wall lesion frequency.

## MATERIALS AND METHODS

A total of 60 third molars extracted for therapeutic purpose were included in the study. All the teeth were evaluated under a stereomicroscope (Leica Wild M3Z, Germany) to ensure the absence of white spot lesions or caries, developmental defects, microfractures and discoloration.^6^All the samples were stored in 0.01% thymol solution^7^ to achieve disinfection and prevent dehydration. Fluoride-free prophylaxis^8^ was done and teeth were stored in double deionized distilled water at room temperature until further use.

The samples were divided into three groups—A, B and C, each containing 20 teeth. On the middle third of buccal surface of each of the third molar tooth, fissure cavity^9^ was prepared using Fissurotomy bur (SS White Burs, Lakewood, NJ) and a high speed handpiece (NSK, PANA AIR) of size 5 × 2 × 1.5 mm without bevel or feather edge preparation. The dimensions of the cavity were measured with a William’s periodontal probe (Hu-Friedy PQW6, USA) to ensure uniformity of the enamel window in all the samples. All the three groups were color coded for identification as per shown in Table 1 and filled with pit and fissure sealants according to manufacturers’ instructions.

**Table 1:** **Table 1: **Color coding of groups along with pit and fissure sealants

*Sr. no.*		*Group*		*Color coding*		*Pit and fissure sealant*	
1.		Group A		Red		Nonfluoridated resin sealant (Helioseal^®^ pit and fissure sealant)	
2.		Group B		Green		Fluoride-releasing resin sealant (Guardian Seal™ pit and fissure sealant)	
3.		Group C		Pink		Glass ionomer pit and fissure sealant (GC Fuji VII™ GI pit and fissure sealant)	

### Application of Sealants to the Cavities

- *Group A (Nonfluoridated pit and fissure sealant; Helioseal^®^ pit and fissure sealant): *The cavity in each sample was acid etched^10^ with 37% phosphoric acid gel for 30 seconds,^11^ rinsed with double deionized distilled water for 10 seconds and dried with oil-free compressed air for 10 seconds. The material was then placed in the cavity of each tooth with the manufacturer’s direct delivery system up to cavosurface margin and light cured for 40 seconds with light curing unit (Bee Cool, Plus Top light – LED light curing unit, Taiwan).
- *Group B (Fluoride-releasing pit and fissure sealant, Guardian Seal™ pit and fissure sealant): *The same procedure as described for group A was followed with respect to the samples belonging to group B.
- *Group C (Glass ionomer pit and fissure sealant, GC Fuji VII™ GI pit and fissure sealant): *The cavity of each sample was cleaned for 15 seconds with cavity conditioner,^12^ rinsed with double deionized distilled water for 10 seconds and dried with oil-free compressed air for 10 seconds. The powder and liquid were mixed according to manufacturer’s instructions and placed into cavities. Excess material was removed with plastic instrument and light cured for 40 seconds (Bee Cool, Plus Top light – LED light curing unit, Taiwan). All the surfaces of molars were coated with acid resistant varnish leaving 1 mm rim of exposed sound enamel surrounding the sealant-filled cavities and subjected to thermocycling^13^ in artificial saliva.^14^

Samples were suspended in acidified gelatin gel^15^ for 6 weeks at 37°C to induce artificial caries like lesion on exposed enamel rim. The acidified gelatin gel was changed at weekly interval, as pH of solution gets altered with time. Each tooth was then cleaned thoroughly with double de- ionized distilled water.

Longitudinal tooth section of 150 μ m thickness was obtained by cutting through the enamel window of tooth using a Silverstone-Taylor hard tissue microtome (Leica SP 1600, Leica Microsystems, Nussloch, Germany). The section was mounted on glass slide and evaluated under polarized light microscope (Leica, Leica Microsystems, Nussloch, Germany).^16^

### Quantification of the Lesions using Leica QWin Software

**Fig. 1:**
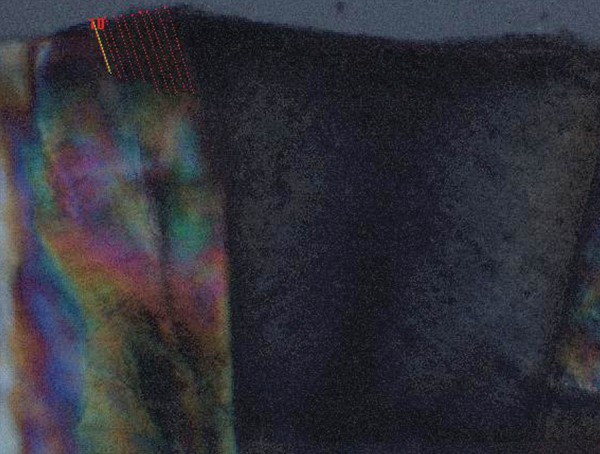
Photomicrograph showing measurement of advancing front of caries like lesions at 10 points

Each section was examined under polarized light microscope and photomicrograph of each section was taken. The mean lesion depths of caries like lesions were determined in a blinded fashion by projecting the photomicrographs onto a computer interfaced digitized tablet and measuring 10 points along the advancing front lesions. Using the same protocol, the presence or absence of wall lesions was determined for each specimen. The advancing front along the body of the outer surface lesion was measured, with the first measurement located 100 micrometers from the cavity preparation (Fig. 1).

For statistical analysis, one-way ANOVA was used for multiple group comparisons followed by post hoc Tukey’s test for groupwise comparison (A *vs *B, A *vs *C, B *vs *C). The results were expressed as mean ± SD, coefficient of variation and range values.

## RESULTS

**Fig. 2:**
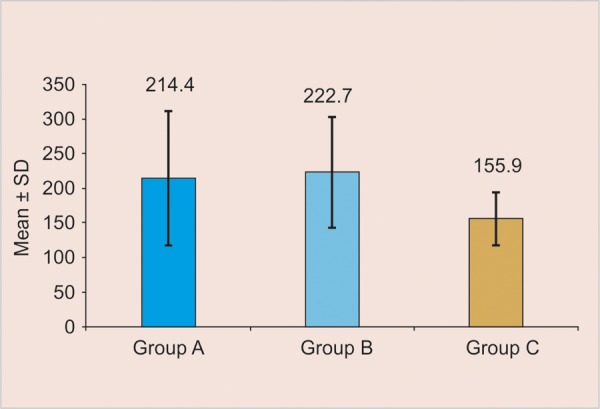
Mean demineralization values of groups A, B and C

Table 2 and Figure 2 show the mean depths of the outer lesions from the three treatment groups. The mean depth of outer lesions was compared using ANOVA and post-hoc Tukey’s test for groupwise comparison (significance level of p < 0.05).

**Table 2:** **Table 2: **Descriptive statistics showing the intergroup comparison of the significance p-values of difference in demineralization among three experimental groups

*Groups*		*Demineralization* *Mean ± SD*		*Difference between groups*						*Significance*
			*Groups compared*		*Mean difference*		p-value*			
Group A		214.44 ± 97.44	A-B		8.29		0.93 NS			p > 0.05
Group B		222.73 ± 80.66	A-C		58.45		0.049 S			p < 0.05
Group C		155.99 ± 37.87	B-C		66.74		0.021 S			p < 0.05

ANOVA F = 4.51; p < 0.05; S: Significant; p > 0.05; NS: Not significant; *: Post-hoc Tukey’s test; SD: Standard deviation

The mean outer lesion depth was the least for group C (glass ionomer pit and fissure sealant) at 73%. The difference in outer lesion depth of group C with that of the remaining groups was found to be significant. As shown in Table 3, the outer lesions adjacent to cavities filled with the conventional nonfluoride-containing sealant had a reduction of 3% in depth when compared with those filled with fluoride-releasing sealant. The outer lesions adjacent to the cavities filled with glass ionomer sealant had a reduction of 27% in depth when compared with the conventional nonfluoride-containing sealant. The glass ionomer sealant group also had a 30% reduction in outer lesion depth when compared with the fluoride-releasing sealant group. The percentage of reduction was calculated by dividing the mean lesion depth for the treatment group by the mean lesion depth for the control group and multiplying the result by 100%.

## DISCUSSION

Pits and fissures are more vulnerable to caries initiation due to variation in shapes, tortuousness with invaginations or irregularities and narrowness (~ 0.1 mm wide). As a result these are ideal sites for the retention of bacteria and food remnants, rendering mechanical means of debridement inaccessible as toothbrush bristle (0.2 mm) is too large to penetrate most of the fissures.^4^

Attempts were made to prevent pit and fissure caries by various means like prophylactic odontotomy, enameloplasty, use of topical and systemic fluorides and various adhesive materials like cements and resins.^4^ Use of pit and fissure sealants was thus conceptualized to prevent initiation of caries in fissures which is conservative modality of caries prevention.^17^ The cariostatic properties of sealants are attributed to the physical obstruction of the pits and fissures preventing colonization of new bacteria and penetration of fermentable carbohydrates, so that remaining bacteria cannot produce acid in cariogenic concentration.^18^ The role of fluoride released from dental materials in the prevention of caries^19^ has been evidenced from *in vitro *and *in vivo *studies, supporting the contention that frequent supply of F^–^ at low concentration decrease the enamel demineralization and accelerates the remineralization process.^20^

Although other agents, such as fluoridated varnishes, dentifrices, mouth rinses and gels can reduce the prevalence of caries, the fluoride released from dental materials also plays a promising role in caries prevention.^21,22^ The ability of a dental material to act as a fluoride reservoir is a distinct advantage in caries resistance, both at the enamel restorative interface and adjacent to the outer enamel surface near the fluoride-releasing dental material.^23,24^ Glass ionomer cement (GIC) is shown to release fluoride slowly over a period of time^25^ into the surrounding enamel yielding cariostatic effects.^26^ Chemical bonding of GIC to enamel and dentin without etching is the additional advantage, making it much easier to handle.^12^ Because of its well-known cariostatic effect, attempts were made consistently for more than 25 years to add fluoride in resin sealants^27^ and efforts to combine the two continue today.^28^

**Table 3:** **Table 3: **Effect of fluoride release from sealant material on enamel demineralization and wall lesion frequency

*Sealant*		*Mean outer lesion depth (micrometers)*		*Wall lesion frequency*		*Reduction in outer lesion depth*
Group A (nonfluoridated pit and fissure sealant)		214.44 ± 97.44		0%		3% when compared with fluoride-releasing sealant
Group B (fluoridated pit and fissure sealant)		222.73 ± 80.66		0%		
Group C (glass ionomer pit and fissure sealant)		155.99 ± 37.87		0%		27% when compared with conventional nonfluoride-containing seal style="border-bottom:solid 1px #000000;"ant 30% when compared with the fluoride- releasing sealant group

In the present study, glass ionomer pit and fissure sealant has shown highest inhibition of demineralization of adjacent enamel. The difference in inhibition of demineralization between glass ionomer pit and fissure sealant and fluoridated and nonfluoridated resin sealant was statistically significant. The result is attributed to F^–^ released from glass ionomer cement by means of three discrete mechanisms: Surface wash off, diffusion through pores and cracks and bulk diffusion.^29^ Similar findings were confirmed by some *in vitro *and *in vivo *studies showing a sustained fluoride release from GIC to the surrounding dental structures^30^ and tooth microenvironment.^31^ Study evaluating GIC as pit and fissure sealants clinically has proved to reduce caries susceptibility.^32^

There was no significant inhibition of demineralization seen between fluoridated as well as nonfluoridated resin sealants thus, confirming previous results.^30,33^

The above findings could be explained by the differences in the composition between ionomeric and resinous materials, resulting in subsequent differences in fluoride releasing profiles.^33^ Diffusion of water into the material is necessary for the formation of hydrogen ions that attack the fluoride-containing glass particles, releasing fluoride. That is why ionomeric materials are more permeable to water, enhancing fluoride diffusion and release.^34^ On the other hand, the matrix of resinous sealants is much less hydrophilic, making fluoride release more difficult.^35^

No wall lesions were found in any of the specimens in this study. Absence of wall lesions may be justified due to reduction of microleakage along the tooth-sealant material interface due to acid etching or conditioning of enamel. Micropores and microprojections are created on enamel surface causing penetration and polymerization of sealants in these areas, forming a mechanical bond with the tooth. Optimal bonding of resin sealants to enamel depends on proper and adequate conditioning of enamel.^36^ The result obtained in this study can be confirmed by other studies showing that the sealants exhibited small or no dye microleakage at the interface between sealant and dental enamel.^37^

Depending on the environment, all pit and fissure sealants may act differently due to other variables like preparation of fissures, enamel etching and conditioning, application of bonding agents and contamination of prepared surfaces of fissures. Appropriate method of application of sealants is also a determining factor to reduce the microleakage thus, reducing wall lesion, which may further lead to formation of secondary caries.

## CONCLUSION

- The inhibition of demineralization in enamel adjacent to glass ionomer pit and fissure sealants was the highest followed by fluoridated and nonfluoridated resin sealants.
- The inhibition of demineralization in enamel adjacent to fluoridated and nonfluoridated resin sealants was comparable.
- No wall lesions were found in any of the specimens in all of the pit and fissure sealants.
